# Exposure to Radiofrequency Electromagnetic Fields and Sleep Quality: A Prospective Cohort Study

**DOI:** 10.1371/journal.pone.0037455

**Published:** 2012-05-18

**Authors:** Evelyn Mohler, Patrizia Frei, Jürg Fröhlich, Charlotte Braun-Fahrländer, Martin Röösli, the QUALIFEX-team

**Affiliations:** 1 Swiss Tropical and Public Health Institute, Basel, Switzerland; 2 University of Basel, Basel, Switzerland; 3 Laboratory for Electromagnetic Fields and Microwave Electronics, ETH Zürich, Zürich, Switzerland; University of Pennsylvania School of Medicine, United States of America

## Abstract

**Background:**

There is persistent public concern about sleep disturbances due to radiofrequency electromagnetic field (RF-EMF) exposure. The aim of this prospective cohort study was to investigate whether sleep quality is affected by mobile phone use or by other RF-EMF sources in the everyday environment.

**Methods:**

We conducted a prospective cohort study with 955 study participants aged between 30 and 60 years. Sleep quality and daytime sleepiness was assessed by means of standardized questionnaires in May 2008 (baseline) and May 2009 (follow-up). We also asked about mobile and cordless phone use and asked study participants for consent to obtain their mobile phone connection data from the mobile phone operators. Exposure to environmental RF-EMF was computed for each study participant using a previously developed and validated prediction model. In a nested sample of 119 study participants, RF-EMF exposure was measured in the bedroom and data on sleep behavior was collected by means of actigraphy during two weeks. Data were analyzed using multivariable regression models adjusted for relevant confounders.

**Results:**

In the longitudinal analyses neither operator-recorded nor self-reported mobile phone use was associated with sleep disturbances or daytime sleepiness. Also, exposure to environmental RF-EMF did not affect self-reported sleep quality. The results from the longitudinal analyses were confirmed in the nested sleep study with objectively recorded exposure and measured sleep behavior data.

**Conclusions:**

We did not find evidence for adverse effects on sleep quality from RF-EMF exposure in our everyday environment.

## Introduction

In the last two decades, emerging wireless technologies like mobile or cordless phones have led to increasing exposure to radiofrequency electromagnetic fields (RF-EMF) in everyday life [1], [2]. As a consequence, public concern about possible health effects due to RF-EMF exposure arose and various representative population surveys in Europe reported that sleep disturbances were the most common health complaints attributed to RF-EMF exposure [3]–[5].

Several randomized, double blind studies addressed the question whether short-term RF-EMF exposure affects sleep measures such as brain activity recorded by means of electroencephalography (EEG). Most of the studies were conducted in a laboratory setting applying well controlled exposure conditions mimicking a mobile phone handset exposure during 30 to 45 minutes [6]–[11]. Overall, these laboratory studies demonstrated fairly consistently that exposure prior to sleep increased the power in the spindle frequency range during sleep stage 2 of the non-REM sleep in the first few hours of sleep. It is unclear whether these changes in sleep EEG indicate adverse health effects or detrimental sleep quality. Interestingly, two studies that observed effects of mobile phone handset exposure on the EEG and that also investigated subjectively rated sleep quality did not find alterations in subjectively rated sleep quality [8], [10]. However, the statistical power of these studies to detect such effects on sleep quality is low because of the small sample size. Moreover, subtle effects on sleep quality may not be observable in an unfamiliar environment of a sleep laboratory with electrodes attached to the head. Epidemiological studies allow for investigating larger populations and are also suitable to address effects of prolonged exposure of several months or even years. So far, no epidemiological study has explored the effect of mobile phone use on sleep using objectively recorded data on mobile phone use provided by network operators. The few studies dealing with self-reported mobile phone use [12] are not reliable as self-reported exposure data in combination with self-reported outcomes are prone to bias [13].

Mobile and cordless phones produce a relatively high exposure to the head but not to the rest of the body as EMF is rapidly decreasing with distance [2]. As a consequence cumulative RF-EMF exposure of a moderate or heavy wireless phone user is dominated by these close to body sources [1]. On the other hand, environmental RF-EMF sources such as mobile phone base stations, broadcast transmitter or W-LAN access points, produce a continuous but lower and more homogenous exposure to the whole body. Interestingly the public is more concerned about health effects from these environmental RF-EMF sources [5], [14]. In response to these public complaints, a few epidemiological studies on sleep quality addressed exposure from mobile phone bases stations [15]–[17]. These studies did not indicate an exposure-response association; however, their reliability is limited due to their cross-sectional design.

Thus, there is an urgent need for a prospective cohort study on sleep quality addressing all aspects of RF-EMF exposure in our everyday life, which includes exposure to environmental far-fields (e.g. mobile phone base stations) and exposure to sources close to the body localized to the head (mobile and cordless phone use). The aim of this study was to investigate a possible association between different objective RF-EMF exposure surrogates and self-reported sleep quality in a large sample (longitudinal study) and to check the consistency of the results in a subsample with measured RF-EMF exposure and measured sleep behavior data (nested sleep study). Main characteristics of these two study components are presented in Table 1.

**Table 1:** Overview on the two study components.

Study characteristics	Longitudinal study	Nested sleep study
Number of participants	955a)	119b)
Outcomes	Written questionnaire:	Actigraphy:
	- daytime sleepiness	- sleep duration
	- sleep disturbances	- sleep efficiency
		Sleep diary:
		- restfulness of sleep
		- wellbeing in the morning
Exposure measures	Written questionnaire:	Personal measurements:
	- mobile phone use	- everyday life exposure to all sources (during one typical working day)
	- cordless phone use	- night-time exposure to all sources in the bedroom
	Operator recorded data:	- fixed site transmitter exposure in the bedroom
	- mobile phone use	
	Modelling:	
	- everyday life exposure to all sources	
	- night-time exposure to all sources in the bedroom	
	- fixed site transmitter exposure in the bedroom	
Type of data analysis	Longitudinal:	Cross-sectional:
	- cohort analysis	- random effect regression models with a 1-day lag autocorrelation term
	- change analysis	

a) After exclusion of nightshift workers (n = 89) and users of sleeping drugs (n = 81).

b) 1 person was excluded because of sleeping drug consumption during all 14 nights.

## Materials and Methods

### Ethics statement

Ethical approval for this study was received from the Ethical Commission of Basel on March 19^th^, 2007 (EK: 38/07). Written informed consent was obtained from the participants of the nested sleep study and of the participants of the longitudinal study for providing the mobile phone operator data.

### Longitudinal study

For the present study, we invited 3763 residents from the Basel area (Switzerland) randomly selected from communal population registries. Eligible participants were between 30 and 60 years old, Swiss residents or people who lived in Switzerland for at least five years. A baseline survey was conducted in May 2008 and the follow-up in May 2009. Information was collected on sleep quality, possible confounders and relevant exposure predictors including use of mobile and cordless phones. Exclusion criteria for the analyses of sleep data presented in this paper were regular usage of sleeping pills and night shift working either at the baseline or follow-up survey.

In the written questionnaire of the baseline and the follow-up questionnaire, we used seven items of the Epworth Sleepiness Scale [18] ranging from 0 (no daytime sleepiness) to 21 (excessive daytime sleepiness) to assess excessive daytime sleepiness. Due to a technical problem in the production of the questionnaire, the eighth question from the Epworth Sleepiness Score was accidentally skipped (“Lying down to rest in the afternoon when circumstances permit”). Sleep disturbances were determined by means of four standardized questions from the Swiss Health Survey 2007 [19]. The four questions asked about the frequency of difficulty in falling asleep, fitful sleep, waking phases during night, and waking too early in the morning using a four-point Likert scale with categories “never”, “rare”, “sometimes” and “most of the time”. All items were added up and a linear score ranging from 0 (no sleep disturbances) to 12 (heavy sleep disturbances) was built.

Due to the unknown mechanism of radiofrequency electromagnetic radiation on biological organisms, we used six different exposure surrogates to assess far field exposure and exposure from sources operating close to the body. With respect to local exposure to the head (close to body exposure), we asked participants in the written questionnaire about their average mobile and cordless phone use per week during the past six months. Informed consent was also sought from participants to obtain their mobile phone connection data for the previous six months of each survey from the three Swiss mobile phone network operators (operator data).

For far field exposure, we used a three-dimensional geospatial propagation model in which average RF-EMF from fixed site transmitters (mobile phone base stations and broadcast transmitters) was modeled for the apartment of each study participant [20]. The model was validated in an independent dataset. Additionally, to predict total personal far-field exposure to all relevant environmental RF-EMF sources, we developed and validated a prediction model [21]. This model is based on the geospatial propagation model and includes additional exposure relevant factors such as housing characteristics (type of house wall and window frame) and behavioral factors (e.g. ownership of a cordless phone or wireless LAN). A separate model was developed to estimate total environmental RF-EMF exposure during night.

### Nested sleep study

From the responders of the baseline cohort survey, 120 participants were selected for a nested sleep study. We did not recruit persons with children less than two years, people who had experienced a long distance flight within the last three weeks, people with severe illnesses, people who regularly consumed sleeping pills and shift workers. We used our exposure prediction model to oversample highly exposed persons to maximize the exposure range in the nested sleep study.

In the participants of the nested sleep study, sleep behavior was measured by means of a wrist actigraphic device (AW7, Cambridge Neurotechnology) with an epoch length of 15 seconds during two weeks. Participants were asked to wear this device on the non-dominant wrist during two weeks and were advised to press an event marker when trying to fall asleep or getting up. They also received a sleep diary, which they had to fill in every morning and every evening. This diary was based on the sleep diary suggested by the German Society of Sleep Medicine (http://www.charite.de/dgsm/dgsm/fachinformationen_frageboegen_schlaftagebuecher.php?language=german) collecting information on waking phases during the night, alcohol and caffeine consumption prior to sleep, and physical activity during the day. The sleep diary also provided backup data for bedtime and getting up time in case participants forgot to press the event marker of the actimeter. In the morning participants rated the restfulness of the sleep using a scale from 1 (very restless sleep) to 5 (very restful sleep) as well as their well-being using a scale from 1 (depressed) to 6 (easygoing).

Actigraphic data were analyzed using the software provided by the manufacturer. A study assistant checked the night data for artifacts and the diary data were systematically used for data quality control. Nights in which participants forgot to wear the actigraphic device were replaced with the data from the sleep diary. We excluded from the data analysis nights during which a switching from daylight saving time to regular time and vice versa took place, nights when participants slept at another place or nights with sleeping pill consumption. Two sleep parameters were extracted from the actigraphic measurements: total sleep duration and sleep efficiency. Definitions of these parameters are given in Table 2.

**Table 2:** Definition and distributions of the sleep quality parameters.

	Parameter	Definition	Data sources	Min.	Median	90^th^ perc.	Max.
**Longitudinal study (n = 955)**	Epworth sleepiness scale (ESS)	Excessive daytime sleepiness	Questionnaire	0	Baseline: 5	10	19
					Follow-up: 4	9	21
	Sleep disturbance score	Difficulties with falling asleep, fitful sleep, waking phases during night and awaking too early in the morning	Questionnaire	0	Baseline: 5	9	12
					Follow-up: 5	8	12
**Sleep study (n = 119)**	Total sleep duration [h]	Time from sleep onset to sleep end excluding waking phases	Actigraphy	4.8	7.1	8.0	9.7
	Sleep efficiency [%]	Percentage of time in bed with the intention to sleep that a person sleeps	Actigraphy	79.0	91.2	95.1	96.9
	Restfulness of sleep	How restful was your sleep? 1 “very restless sleep” to 5 “very restful sleep”	Sleep diary	2.8	4	4.6	5
	Well-being in the morning	How do you feel now? 1 “depressed” to 6 “easygoing”	Sleep diary	1.5	4.5	5.8	6

For the sleep study, all estimates are given for the level of the individual (i.e. average over two weeks).

Exposure to all relevant sources of radiofrequency electromagnetic fields was measured with the EME SPY120 (Satimo, Courtaboeuf, France). Exposure measures were taken every 90 seconds during the first week of the measurement period (two weeks). The exposure meter device (exposimeter) was placed in the sleeping room near the bed and the head of the participants. During one typical working day participants were requested to wear the exposimeter to estimate their daytime exposure. Mean exposure values were calculated for measurements in the sleeping room during the night, for fixed site transmitter measurements in the sleeping room and for measurements during the day on which the exposimeter was carried around. Mean values were calculated using regression on order statistics, which allows for nondetects [22]. Missing exposure measurements occurred due to technical problems in 6 participants and 29 participants did not have daytime measurements. Those missing values were replaced with data from the prediction model [21], night-time measurements were replaced with the prediction model for night exposure and exposure to fixed side transmitters was replaced by values of the geospatial propagation model [20].

### Statistical analyses

In the longitudinal study, the association between exposure and outcome was calculated by means of linear regression models. We conducted two different analyses: I) A cohort analysis, where we assessed the association between exposure at baseline and the change in self-reported sleep quality within one year. Three exposure categories were defined a priori for each exposure metric: <50^th^ percentile, 50^th^ to 90^th^ percentile, >90^th^ percentile. II) A change analysis, where we examined whether the change in exposure between baseline and follow-up resulted in a change in self-reported sleep quality. For the change analysis we compared the participants with the 20% largest exposure increase and decrease between baseline and follow-up survey with all other participants who experienced a smaller or no change of exposure between baseline and follow-up survey (reference group). All models were adjusted for age, sex, body mass index, stress level, physical activity per week, smoking status, alcohol consumption, education level, marital status, degree of urbanity, belief in health effects due to RF-EMF exposure, noise annoyance and for moving house between the two surveys. About 20% of the participants in each survey reported to be electro-hypersensitive (EHS) or reported that they thought that they developed detrimental health symptoms due to electromagnetic pollution in everyday life [23]. All models were thus tested for interaction between EHS status and the exposure measures in order to evaluate whether EHS individuals are differently affected by RF-EMF exposure.

In the nested sleep study, we used a random intercept mixed regression model with an autocorrelation term of one-day lag to analyze the association between sleep measures and RF-EMF exposure. All models were adjusted for sex, age, smoking status, body mass index, weekday, percent fulltime equivalent, educational level, presence of a bed partner, weekday and the diary-based variables bedtime, alcohol intake within 4 hours before going to bed, physical activity during the day, and sleeping during the day (more information on the confounders is given in the footnote in Table 3). We built three exposure categories: <median (reference group), 50^th^–90^th^ percentile, >90^th^ percentile.

**Table 3:** Change of sleep duration (in hours) and sleep efficiency (in %) (95%-confidence interval (CI)) for various exposure measures from the nested sleep study.

		Linear multilevel modela)			
	Exposure range [mW/m^2^]	n (individuals)b)	n (nights)	Coeff.	(95%-CI)
**Total sleep duration in h**					
**Total everyday life exposure**					
<median	0.00 to 0.11	60	777	0.00	
50.–90. percentile	0.11 to 0.42	48	616	0.07	(−0.18;0.32)
>90. percentile	0.45 to 16.69	11	158	0.19	(−0.21;0.60)
**Night-time exposure**					
<median	0.00 to 0.03	60	763	0.00	
50.–90. percentile	0.03 to 0.12	48	624	0.16	(−0.09;0.41)
>90. percentile	0.12 to 2.18	11	164	0.16	(−0.24;0.56)
**Fixed site transmitter**					
<median	0.00 to 0.01	60	778	0.00	
50.–90. percentile	0.02 to 0.06	48	622	0.07	(−0.17;0.32)
>90. percentile	0.08 to 1.39	11	151	0.00	(−0.43;0.43)
**Sleep efficiency in percent**					
**Total everyday life exposure**					
<median	0.00 to 0.11	60	777	0.00	
50.–90. percentile	0.11 to 0.42	48	616	1.21	(−0.02;2.44)
>90. percentile	0.45 to 16.69	11	158	0.43	(−1.54;2.41)
**Night-time exposure**					
<median	0.00 to 0.03	60	763	0.00	
50.–90. percentile	0.03 to 0.12	48	624	0.80	(−0.41;2.01)
>90. percentile	0.12 to 2.18	11	164	−0.67	(−2.60;1.27)
**Fixed site transmitter**					
<median	0.00 to 0.01	60	778	0.00	
50.–90. percentile	0.02 to 0.08	48	622	0.80	(−0.40;1.99)
>90. percentile	0.10 to 1.40	11	151	−1.04	(−3.11;1.02)

a) adjusted for: age, percent fulltime equivalent, bedtime (derived from diary) (all linear), sex, body mass index (<25, ≥25), smoking status, weekday (weekend vs. workday), presence of a bed partner, alcohol intake within 4 hours before going to bed (diary), physical activity during the day (diary), sleeping during the day (diary) (all binary), and educational level (3 categories).

b) The division into the exposure categories was done on the individual level.

All statistical analyses were carried out using STATA 10.1 (StataCorp, College Station, TX, USA).

## Results

### Study population

In total, 1375 participants filled in the baseline questionnaire in 2008 and 1125 subjects filled in the follow-up questionnaire one year later (response rate 82%). 170 participants were excluded from the longitudinal analyses due to night shift working (89 participants) and consumption of sleeping pills (81 participants). The analyses of our longitudinal study were therefore performed with 955 subjects. Detailed information on the characteristics of the study participants are described in Table 4. Average age of the participants was 47 years. Generally, characteristics of the study participants in the baseline and follow-up survey were comparable [24]. Health status was generally good in all participants.

**Table 4:** Characteristics of the study participants of the longitudinal study at follow-up (baseline data are presented in Mohler et al. 2010 [24]) and of the participants of the nested sleep study.

	*Longitudinal study (n = 955)*	%	*Nested study (n = 119)*	*%*
Age (years)				
30–40	224	24	*26*	*22*
41–50	329	34	*36*	*30*
51–60	402	42	*57*	*48*
Sex				
Female	578	61	*73*	*61*
Male	377	39	*46*	*39*
Health statusa)				
Very good	323	34	*45*	*38*
Good	530	56	*64*	*54*
Half-half	83	9	*10*	*8*
Bad	8	1	*0*	*0*
Very bad	1	<1	*0*	*0*
Educational levela)				
None	51	5	*2*	*2*
Apprenticeship	456	48	*60*	*50*
Higher education/University	448	47	*57*	*48*
Self-reported electromagnetic hypersensitivity^a^^,^ b)				
Yes	195	20	*23*	*19*
No	760	80	*96*	*81*
Owning a mobile phonea)				
Yes	909	95	*107*	*90*
No	41	4	*12*	*10*
Owning a cordless phonea)				
Yes	800	84	*87*	*73*
No	150	16	*32*	*27*
Owning wireless LANa)				
Yes	390	41	*57*	*48*
No	558	59	*62*	*52*

a) Data may not sum up to 100% due to missing data.

b) Answering yes to either “Are you electro hypersensitive?” or “Do you think that you develop detrimental health symptoms due to electromagnetic pollution in everyday life?”

In the nested sleep study, age and gender distribution were comparable with participants of the longitudinal study (Table 4). Twenty-two percent of the participants of the nested sleep study lived alone, 48% with a partner and 30% with children. Sleeping data for 1680 nights were collected from 120 participants. One person was excluded from all analyses due to sleeping drug consumption during all 14 nights. At the time of recruitment, this person did not state that he/she regularly took sleeping pills. Additionally, a total number of 115 nights were excluded from data analyses because participants did not sleep in their own house (77 nights), and/or due to clock change (16 nights), and/or due to sleeping pill consumption (10 nights) and/or because both actigraphic measurements and sleep diary data were missing (18 nights).

### Exposure to RF-EMF

Table 5 shows the ranges of the RF-EMF levels in all exposure categories of the various exposure metrics for the longitudinal study at baseline (cohort analysis) and the changes between baseline and follow-up survey (change analysis). At baseline self reported arithmetic mean mobile phone use was 61.6 minutes per week. Arithmetic mean cordless phone use was 73.8 minutes per week. For the subset of 389 study participants who consented to provide operator recorded connection data, recorded arithmetic mean duration of mobile phone use was 26.4 minutes and self-reported mobile phone use was 47.7 minutes per week. Time-weighted arithmetic mean RF-EMF exposure at baseline was 0.12 mW/m^2^ for everyday life exposure, 0.02 mW/m^2^ for fixed site transmitters and 0.01 mW/m^2^ during night.

**Table 5:** Exposure ranges of the longitudinal study for all study participants (n = 955): ranges in power flux densities to different exposure sources for all included study participants at follow-up survey and the change in exposure levels between baseline and follow-up.

	Exposure at baseline		Change (between baseline and follow-up)	
Close to body exposure				
**Mobile phone use [h/week]**	<Median	0.00 to 0.23	Decrease	−11.67 to −0.15
	50th–90th percentile	0.23 to 3.50	No relevant change	−0.13 to 0.15
	>90th percentile	3.50 to 17.5	Increase	0.15 to 17.50
**Operator data** a **[h/week]**	<Median	0.00 to 0.15	Decrease	−2.85 to −0.18
	50th–90th percentile	0.16 to 1.30	No relevant change	−0.17 to 0.04
	>90th percentile	1.33 to 8.61	Increase	0.04 to 1.49
**Cordless phone use [h/week]**	<Median	0.00 to 0.35	Decrease	−9.27 to −0.58
	50th–90th percentile	0.93 to 4.67	No relevant change	−0.35 to 0.58
	>90th percentile	9.33 to 9.33^b^	Increase	0.87 to 9.33
Far field exposure				
**Total exposure [mW/m^2^]**	<Median	0.00 to 0.12	Decrease	−0.14 to −0.02
	50th–90th percentile	0.12 to 0.17	No relevant change	−0.02 to 0.03
	>90th percentile	0.17 to 0.41	Increase	0.03 to 0.18
**Exposure during night [mW/m^2^]**	<Median	0.00 to 0.00	Decrease	−0.23 to −0.00
	50th–90th percentile	0.00 to 0.04	No relevant change	−0.00 to 0.00
	>90th percentile	0.05 to 0.40	Increase	0.00 to 0.23
**Residential exposure through fixed site transmitters [mW/m^2^]**	<Median	0.00 to 0.01	Decrease	−0.16 to −0.00
	50th–90th percentile	0.01 to 0.05	No relevant change	−0.00 to 0.00
	>90th percentile	0.05 to 1.43	Increase	0.00 to 0.62

For the change analysis we compared the participants with the 20% largest exposure increase and decrease between baseline and follow-up survey with all other participants, who experienced a smaller or no change of exposure between baseline and follow-up survey (no relevant change).

a) n = 389 at baseline (cohort analyses) and n = 245 at follow-up (change analyses).

b) equal values due to the use of categories in the questionnaire.

Measured exposure levels of the nested sleep study are presented in Table 3. Measured arithmetic average exposure in the sleeping room during the night was 0.11 mW/m^2^. Average exposure to fixed site transmitters in the sleeping room was 0.08 mW/m^2^. Arithmetic mean measured daytime exposure during a typical working day was 0.35 mW/m^2^.

### Self reported sleep quality (longitudinal study)

**Figure 1:**
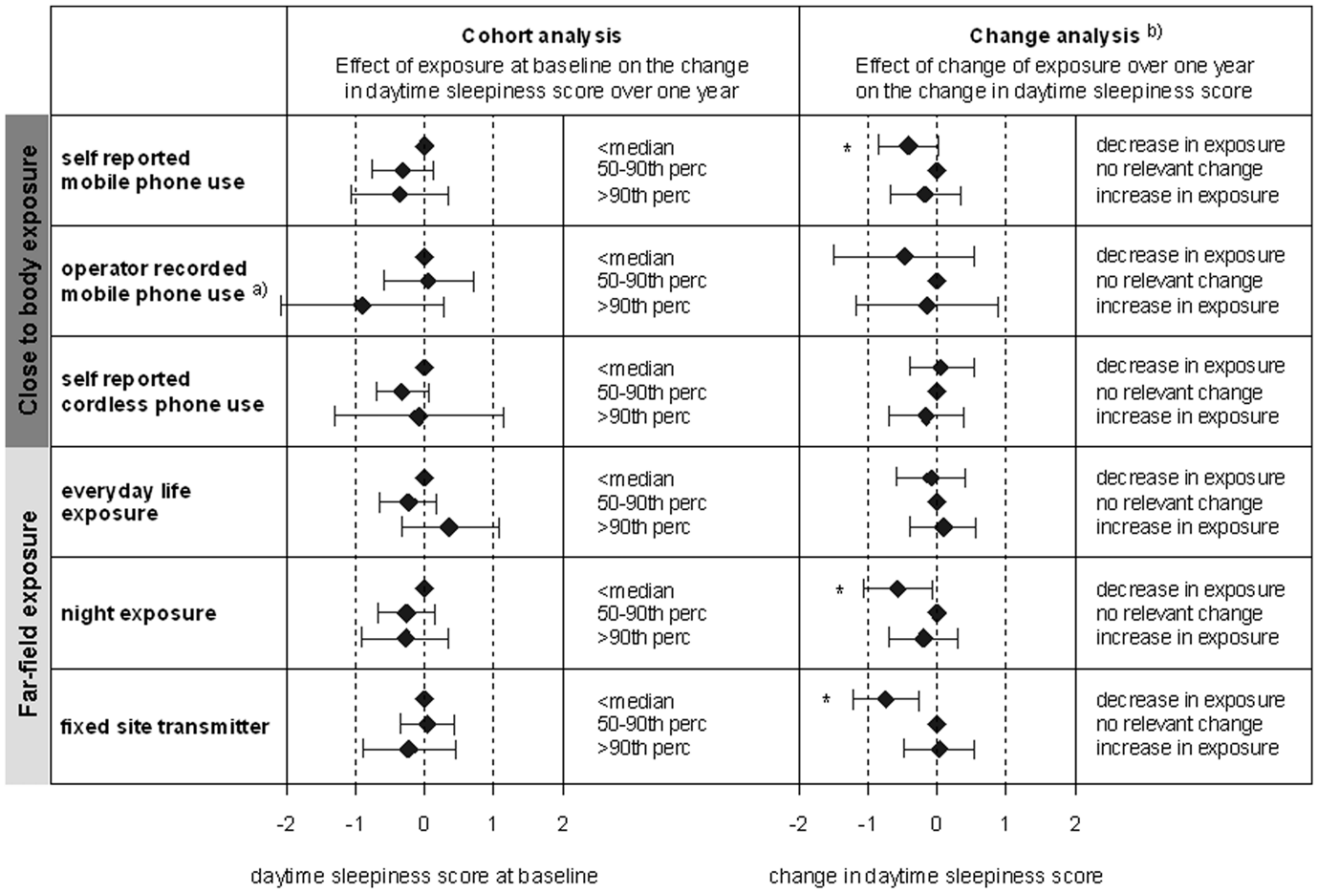
Results of the longitudinal analysis on daytime sleepiness score: Diamonds refer to the change in sleep score and the horizontal lines mark the 95% confidence intervals. An increase in score refers to an increase in daytime sleepiness. * indicates statistical significance. All models are adjusted for age, body mass index, stress level, physical activity, noise annoyance (all linear), sex, alcohol consumption, belief in health effects due to RF-EMF exposure, smoking status, degree of urbanity, moving house between the two surveys (all binary), educational level, marital status (categorical). ^a)^ for a subsample of 363 (225) subjects who consented that we receive data from the operator at baseline (follow-up). ^b)^ In the change analysis a decrease and increase in exposure refers to the participants with the 20% largest exposure decrease and increase between baseline and follow-up survey. No relevant change includes all other participants, who experienced a smaller or no change of exposure (reference group).

**Figure 2:**
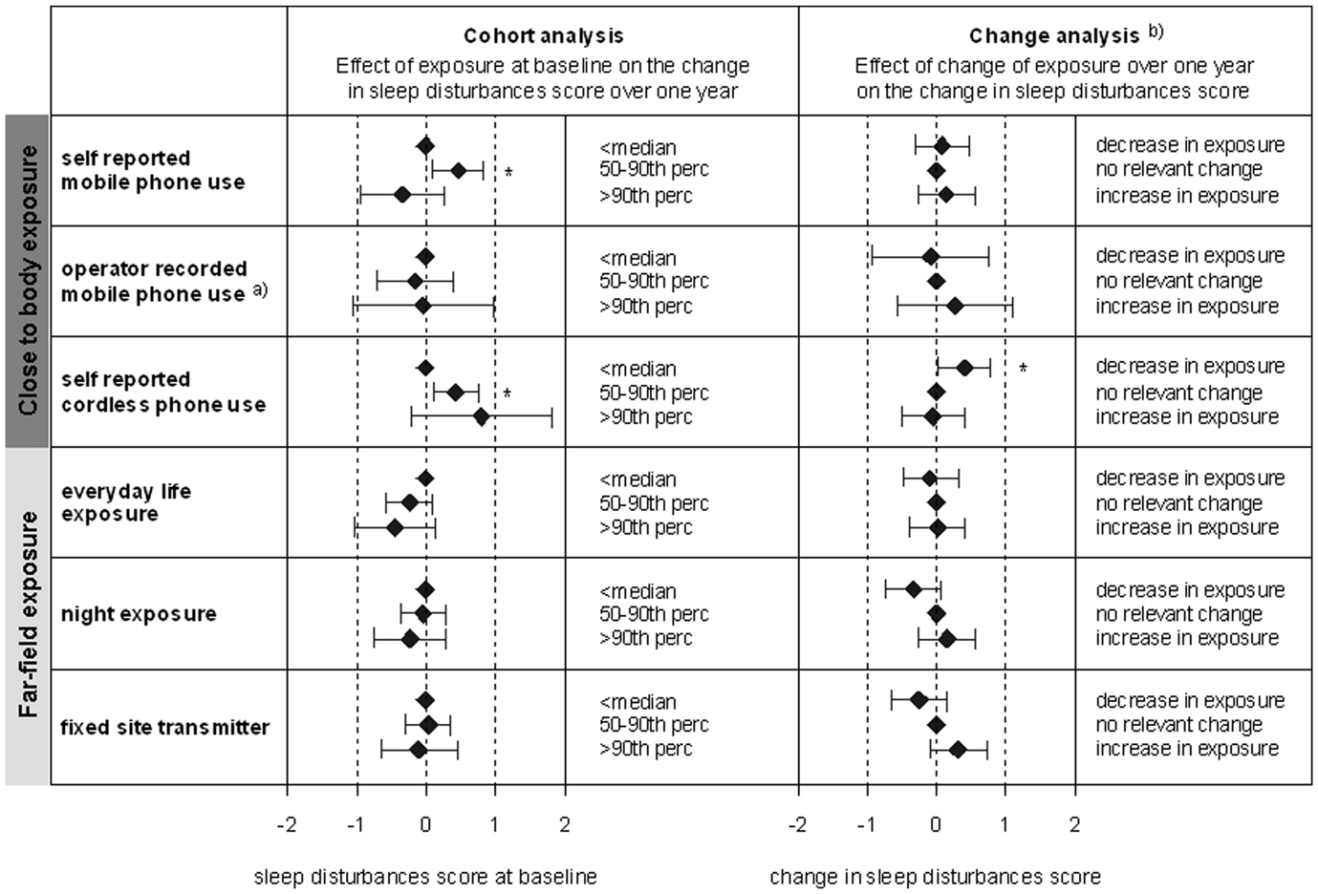
Results of the longitudinal analysis sleep disturbances: Diamonds refer to the change in sleep score and the horizontal lines mark the 95% confidence intervals. An increase in score refers to an increase in sleep disturbances. * indicates statistical significance. Confounders see Fig. 1. ^a)^ for a subsample of 378 (235) subjects who consented that we receive data from the operator at baseline (follow-up). ^b)^ In the change analysis a decrease and increase in exposure refers to the participants with the 20% largest exposure decrease and increase between baseline and follow-up survey. No relevant change includes all other participants, who experienced a smaller or no change of exposure (reference group).

Median daytime sleepiness and sleep disturbances scores per individual at baseline and follow-up are presented in Table 2. The results of the longitudinal analyses on daytime sleepiness are presented in Figure 1 and the results on self-reported sleep disturbances in Figure 2. Overall, six out of 48 effect estimates for the six exposure metrics reached statistical significance. These significant effects concerned different exposure surrogates and outcomes. There was neither a consistent increase in self-reported daytime sleepiness or sleep disturbances if exposure at baseline was high, nor was a change in RF-EMF exposure consistently accompanied by a corresponding change in daytime sleepiness. Generally, interaction testing did not yield a difference in development of sleep disturbances and daytime sleepiness of EHS and non-EHS individuals (data not shown).

### Sleep behavior (nested sleep study)

Measured arithmetic mean sleep duration per individual was 6.9 hours (h) during weekdays (range: 4.9 h to 9.4 h) and 7.8 h during weekends (range: 4.5 h to 11.9 h) (Table 2). Sleep efficiency was on average 91.0% (range: 79.0% to 96.9%) and did not differ statistically significantly between weekdays and weekends. Mean sleep duration (7.1 h vs. 7.2) and mean sleep efficiency (91.0% vs. 91.9%) were similar for actigraphic measurements and self-reports. In Table 3, results of the regression analyses for sleep duration and sleep efficiency are presented for the three measured exposure surrogates. Neither typical everyday exposure to all RF-EMF sources, nor night-time exposure, nor exposure from fixed site transmitters was significantly associated with sleep duration or sleep efficiency. Additionally, we investigated whether RF-EMF exposure was related to self-reported restfulness of sleep as rated each morning in the sleep diary. For all three exposure measures, restfulness of sleep in the participants in the top exposure decile was not significantly altered compared to the reference category: change in score for total everyday exposure was 0.14 units (95% confidence interval: −0.13 to 0.41), for night-time exposure 0.06 units (95% CI: −0.20 to 0.32) and for exposure to fixed site transmitters −0.04 units (95% CI: −0.33 to 0.25). Similarly, well-being in the morning was not related to any of the RF-EMF exposure surrogates (data not shown).

## Discussion

This study did not find indications for an association between typical levels of RF-EMF exposure in an everyday environment and self-reported sleep disturbances or excessive daytime sleepiness considering an exposure period of one year. These results were confirmed in a subsample of 119 study participants with data on sleep behavior measured with actigraphic devices and measured RF-EMF exposure.

### Strength and limitations

To the best of our knowledge this is the first longitudinal study investigating the association between RF-EMF exposure and self-reported sleep quality in a large population sample using objectively recorded exposure data and data on sleep behavior measured with actigraphic devices. The cohort design allows for more robust conclusions, particularly because participation rate in the follow-up survey was rather high (82%). Therefore, in the present cohort and change analyses of the longitudinal study selection bias is expected to be of minor concern.

We applied a comprehensive exposure assessment method. All RF-EMF sources relevant in our everyday environment are included in the model and also personal exposure relevant behaviors are considered. The prediction models of the longitudinal study are based on extensive measurements with personal dosimetric devices. For the development of these prediction models we used weekly measurements of 166 persons and conducted a validation study by repeating the exposure measurements in 31 study participants 21 weeks later on average. In this validation study agreement between personal measurements and the prediction model for everyday exposure was found to be good (Spearman rank correlation: 0.75 (95%-CI 0.53–0.87), sensitivity: 0.67 and specificity 0.96) [21]. To consider the impact of close to body sources, we included self-reported mobile and cordless phone use as well as objective information on mobile phone use from participants who gave their informed consent. Three Swiss mobile phone network operators provided this information. Additionally, we were able to verify our results of the longitudinal analyses with measured data on sleep behavior and environmental RF-EMF exposure in the nested sleep study.

The subjective sleep parameters in the longitudinal study might be considered a weakness of this study. However, we used standardized questions to assess daytime sleepiness and sleep disturbances. Subjectively perceived sleep quality is an established factor influencing personal well-being and is thus health relevant [25]. Alternatively, polysomnographic records could have been used to obtain sleep measures. However, this method may have affected sleep quality of the participants and we were also concerned that such a demanding task for study participants could have created considerable selection bias by attracting mainly persons who are concerned about EMF exposures. As a consequence we used actigraphy, a more convenient tool for study participants, to collect measured data on sleep behavior in the nested sleep study. With these data we could confirm the results of the longitudinal analysis.

With respect to self-reported outcome measures, information bias may be of concern if study participants are aware of their exposure status. For instance, individuals who consider themselves as exposed to mobile phone base station radiation may claim to suffer more often from sleep disturbances. There is some evidence from laboratory trials that more symptoms are reported in open provocations where participants were aware about the exposure status than in subsequent double blind provocations [26]–[28]. However, we could demonstrate in our study that self-estimated RF-EMF exposure to far-field environmental sources is not correlated to objective exposure measured with an exposimeter [29]. Thus, our self-reported outcomes are most likely not affected by information bias.

### Interpretation

We did not find an association between self-reported sleep quality and prolonged exposure to RF-EMF. Our findings are in line with results of cross-sectional surveys about RF-EMF exposure and self-reported sleep quality, which used spot measurements to assess exposure [15], [16], 24 h personal measurements [17], or applied a double blind field experiment with mobile phone base stations [17], [30]. Spot measurements have been shown to be an appropriate exposure proxy [29], but, in contrast to our study, not all relevant sources and only exposure at home is measured. In particular, exposure from mobile phone handsets is not considered. This is a relevant exposure source for a sleep study since it is the most relevant exposure source for the head and various randomized trials found increased power in the spindle frequency range if study subjects were exposed to mobile phones prior to sleep [8]–[10]. This is the first epidemiological study on sleep quality using operator recorded mobile phone use and not only self-estimated exposure data.

We conducted a large number of analyses because in the absence of a known biological mechanism in the low dose range, it was unclear which aspect of exposure might be relevant for sleep disturbances, if any at all. We simultaneously took into account exposure from sources close to the body, producing high, localized and short-term exposures, as well as sources further away, which typically cause lower, more homogenous long-term exposures. Since mobile phone base stations are the EMF source people in Switzerland are most concerned about [5], we wanted to consider the effect of exposure to fixed site transmitters separately. We did not apply a formal multiple endpoint correction (e.g. Bonferroni correction). Instead we checked the consistency and biological plausibility of similar analyses.

Given the absence of an observed association, non-differential exposure misclassification may be of concern. For such ubiquitously distributed exposure sources, some exposure misclassification is unavoidable although we have put considerable effort in validating our methods. Non-differential exposure misclassification is expected to shift the regression coefficients towards zero if there is a true association. Nevertheless, assuming there is a true association, we would expect to see a non-significant exposure-response pattern consistently pointing towards an association. However, this was not observed in our study neither in the direction of a harmful nor in the direction of a beneficial effect. For interpretation of this and similar studies on symptoms, a “healthy communicator effect” may be relevant. Healthy communicator effect refers to the possibility that healthy people may use more often wireless communication devices and thus may be more exposed than ill people. It can thus be considered an analogy to the well known healthy worker effect.

In our study we observed relatively low far-field exposure levels. The levels were far below current standard limits [31] but representative for the RF-EMF exposure situation in the years 2007–2009 in an urban and suburban environment. Also the changes in exposure levels between baseline and follow-up survey were relatively small. Therefore, we are only able to draw conclusions about consequences of small exposure levels and changes, respectively.

We found no evidence that individuals who reported to react sensitively to EMF (electromagnetic hypersensitivity) were more vulnerable to RF-EMF exposure than the rest of the population. This is in line with reported randomized double blind provocation studies addressing short term effects [13], [32]. However, observational research in EHS individuals is limited if one assumes that EHS individuals tend to avoid EMF exposure. If such an intentionally achieved exposure reduction results in a better health status, it could either be mediated by a biophysical mechanism or by a pure nocebo mechanism. In our study, however, we did not observe such changes.

Our longitudinal study captured a latency period of one year. It is not clear whether such a period is sufficient for sleep effects to manifest. Thus, we cannot completely rule out that our study has missed sleep effects that occur after prolonged exposure duration. However, most individuals who reported sleep disturbances in relation to mobile phone base station exposure claimed that such symptoms have occurred within a few days or weeks after a new exposure source was put into operation [33]. Such an effect should have been observable with our study design.

### Conclusion

Overall, we did not find an association between self-reported sleep quality and everyday RF-EMF levels from various sources over one year. By applying a longitudinal design and using objective exposure and measured outcome data, this study increases evidence for the true absence of an effect of everyday RF-EMF exposure on sleep quality.
